# Let it be: fate of a lost closure device

**DOI:** 10.1093/eurheartj/ehab046

**Published:** 2021-02-05

**Authors:** K E Juhani Airaksinen, Juha Lund, Antti Saraste

**Affiliations:** Heart Centre, Turku University Hospital and University of Turku, POB 52, FI-20521 Turku, Finland

A 76-year-old man with a history of stroke and gastrointestinal bleedings without anticoagulation (CHA_2_DS_2_VASc 7, HASBLED 4) was referred to left atrial appendage closure in 2013. Left atrial appendage was tapering and shallow but closure was deemed adequate with a 28 mm Amplatzer cardiac plug (*Panel A*). Adequate position was confirmed with echocardiography and X-ray before discharge. At 4-month control, the patient had been asymptomatic, but closure device had embolized to aortic arch. Computed tomography angiography (*Panel B*) or ultrasound (*Panel C*) images of the aortic arch did not show signs of device thrombosis and blood flow was laminar on both sides of the plug. Percutaneous removal of device was not possible because of severe atherosclerosis and tortuosity of femoral and iliac arteries and surgery was deemed too risky. The patient was treated with aspirin only until 2018 when he had transient ischaemic attack. The position of cardiac plug was unchanged with no thrombi, but a severe stenosis of left carotid artery was found and treated with endarterectomy. Patient recovered with no permanent neurological deficits. Aspirin was changed to apixaban by neurologists and there have been no ischaemic or bleeding events until the end of 2020. This case demonstrates that when an embolized closure device is not in a flow-limiting position in aorta, it may not cause long-term risk for ischaemic or thromboembolic complications and it is not necessary to proceed to risky surgery to remove the device.

**Conflict of interest:** The authors have submitted their declaration which can be found in the article Supplementary Material online.

**Figure:**
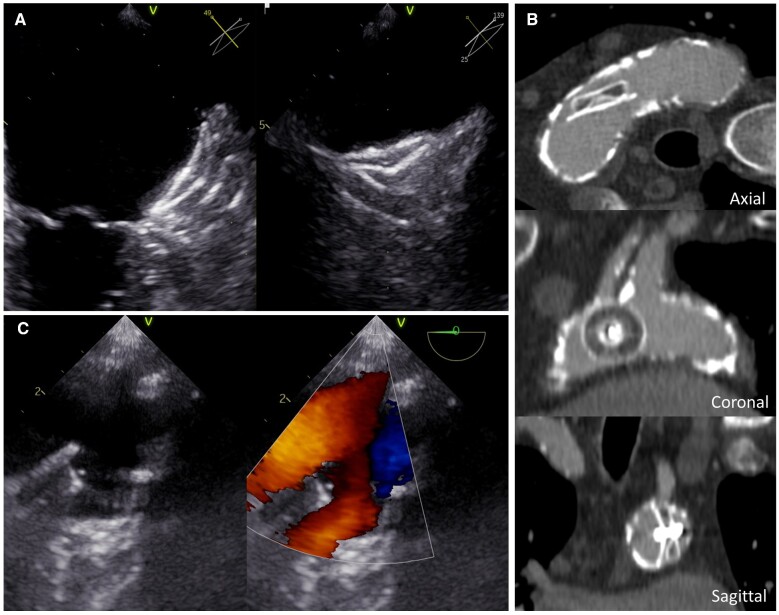


## Supplementary Material

ehab046_Supplementary_Data

