# Supplementary material for: Let it be: fate of a lost closure device
Source: Eur Heart J. 2021 Feb 5;43(13):1363. doi: 10.1093/eurheartj/ehab046 (PMC10016809; doi:10.1093/eurheartj/ehab046)
Supplement: ehab046_Supplementary_Data [file ehab046_supplementary_data.docx]

“**Let it be – Fate of a lost closure device**“ :

All authors have contributed significantly to the submitted work, have read and approved submission of the manuscript, and the manuscript has not been published and is not being considered for publication elsewhere in whole or in part in any language. The authors do not have any commercial associations, consultancies, stock ownerships or other equity interests or patent licensing arrangements that might pose a conflict of interest in connection with the submitted article.
